# Mid- to long-term results of modified non-vascularized allogeneic fibula grafting combined with core decompression and bone grafting for early femoral head necrosis

**DOI:** 10.1186/s13018-020-1565-3

**Published:** 2020-03-24

**Authors:** Chen Changjun, Li Donghai, Zhao Xin, Chen Liyile, Wang Qiuru, Kang Pengde

**Affiliations:** Department of Orthopaedics, West China Hospital, Sichuan University, 37# Guoxue Road, Chengdu, 610041 People’s Republic of China

## Abstract

**Purpose:**

The aim of this study was to determine mid-and-long term follow-up results of patients with early femoral head osteonecrosis who were treated by modified free vascularized fibular grafting combined with core decompression and bone grafting.

**Methods:**

Forty-four patients at early ONFH were included in this study. Visual analog scale (VAS) pain scores, range of hip motion (ROM), and Harris hip score (HHS) were recorded to assess the clinical outcome; Western Ontario McMaster Osteoarthritis index (WOMAC) scores and Short Form 36 health survey (SF-36) were conducted to measure the living quality; X-ray film or magnetic resonance imaging (MRI) was used to evaluate radiographic progression; survivorship was defined as patients did not undergo the total hip arthroplasty (THA) or fusion at the last follow-up. Median follow-up was 7.4 years (6–8.2 years).

**Results:**

The mean VAS score, ROM, and HHS were significantly improved at the final follow-up compared with preoperative values (*p* < 0.001). Health assessment including WOMAC scores and SF-36 were also better than those preoperatively (*p* < 0.001). Seven patients progressed to Ficat III and the four patients progressed to Ficat IV with osteoarthritis. Eight patients who cannot tolerate the pain and had poor living quality underwent THA.

**Conclusion:**

Modified non-vascularized allogeneic fibula Grafting combined with core decompression and bone grafting could improve the clinical outcomes and enhance the quality of life for patients with early ONFH.

## Introduction

Osteonecrosis of the femoral head (ONFH), characterized by reduced local blood flow, death of the osteocytes, and the bone marrow, is a frequently disease occurring in young individuals, which can lead to a progressive destruction of bone architecture, subchondral fracture, extensive hip pain, and loss of joint function [1]. And most of the patients usually progress to the collapse of the femoral head within a mean time of 2 years and even severe secondary symptomatic hip arthritis, as a result, they have to require total hip arthroplasty (THA) to restore joint function [2, 3] . Furthermore, THA is not the best option for young patients because prosthetic replacements rarely last for their lifetime and the associated complications might lead to revision surgery [4]. Thus, effective measures should be taken to treat ONFH as early as possible.

Until now, the optimal treatment for patients with symptomatic early-stage osteonecrosis of the femoral head is still controversial. Normally, conservative treatment and operation are the main methods that we can choose. And some comparatively non-operative methods, such as extracorporeal shock waves, have appeared to be effective in the short term, but the long-term effect is unknown [5]. Actually, most patients still have to refer to operation for further treatment, and operation methods are various, ranging from core decompression, fibular, or iliac bone grafting to Tantalum rod implantation [6–9]. Other methods to protect the femoral head including trans-trochanteric rotational osteotomy, which is aimed to move the affected areas of the femoral head away from the main weight-bearing regions of the joint; however, the treatment effects were still controversy after studied for ages [10, 11]. Furthermore, core decompression combined with tantalum rod placement or fibular grafting is also an effective measure to support the subchondral architecture with a comparatively good success rate, but little bone ingrowth and insufficient mechanical support of subchondral bone can still be seen in some cases [12, 13]. On the other hand, though some disadvantages of vascularization bone grafting have been reported, such as increased measurable but acceptable complication and morbidity risk [14], vascularized fibular grafts was still advocated for most surgeons for vascularization of the graft could enhance its incorporation to the decompression channel, maintain graft viability, and provide perfusion the osteonecrotic area [15–17]. And it has been proved that free vascularized fibular grafting can improve vascularity compared with core decompression in the treatment of femoral head osteonecrosis, which is effective on pain-relieving and function recovering [9]. However, considering the complexity of free vascularized fibula graft, impracticality for community hospitals, and high costs of tantalum rod implantation, an effective and inexpensive method should be found for some less affluent population to delay the progression of ONFH and protect the femoral head before it collapses.

Promoting the repair of defects and improving mechanical support are two significant points. Core decompression with necrosis bone removed causes the bone defects and increases the risk of fracture for lacking the support structures to the subchondral bone area. Thus, in this study, we try to improve the blood supply, protect the mechanical property, and reduce the complexity of the procedures by using modified non-vascularized allogeneic fibula grafting combined with core decompression and bone grafting to treat early ONFH, and evaluate its clinical results, living quality, radiographic outcomes, and survivorship at mid- to long-term follow-up.

## Patients and methods

The study was approved by the Clinical Trials and Biomedical Ethics Committee of West China Hospital, and written informed consent was obtained from all participants. According to the Ficat classification of ONFH, patients whose stage were at Ficat II of ONFH were included in our study. And patients whose previous drug therapy, disease, or surgery histories might influence the outcomes were excluded from the study. Between 2009 and 2011, 50 patients younger than 50 years of age with early ONFH were operated on using the authors’ technique and 44 were available for review in the current study. The diagnosis of osteonecrosis of the femoral head was mainly plain radiographs and confirmed with magnetic resonance imaging. The patients’ background and details are listed in Table 1.

**Table 1:** Demographic characteristic of the patients

Parameters	Median (range)
Total number	44 patients (44 hips)
Age (years)	36. 46 (26–47)
Body mass index (kg/m^2^)	23.8 (18.8–27.6)
Gender (male/female)	29/15
Etiology	
Alcohol-induced	26
Steroid-induced	15
Idiopathic	3
Classification	
Ficat II	44

### Surgical technique

**Fig. 1:**
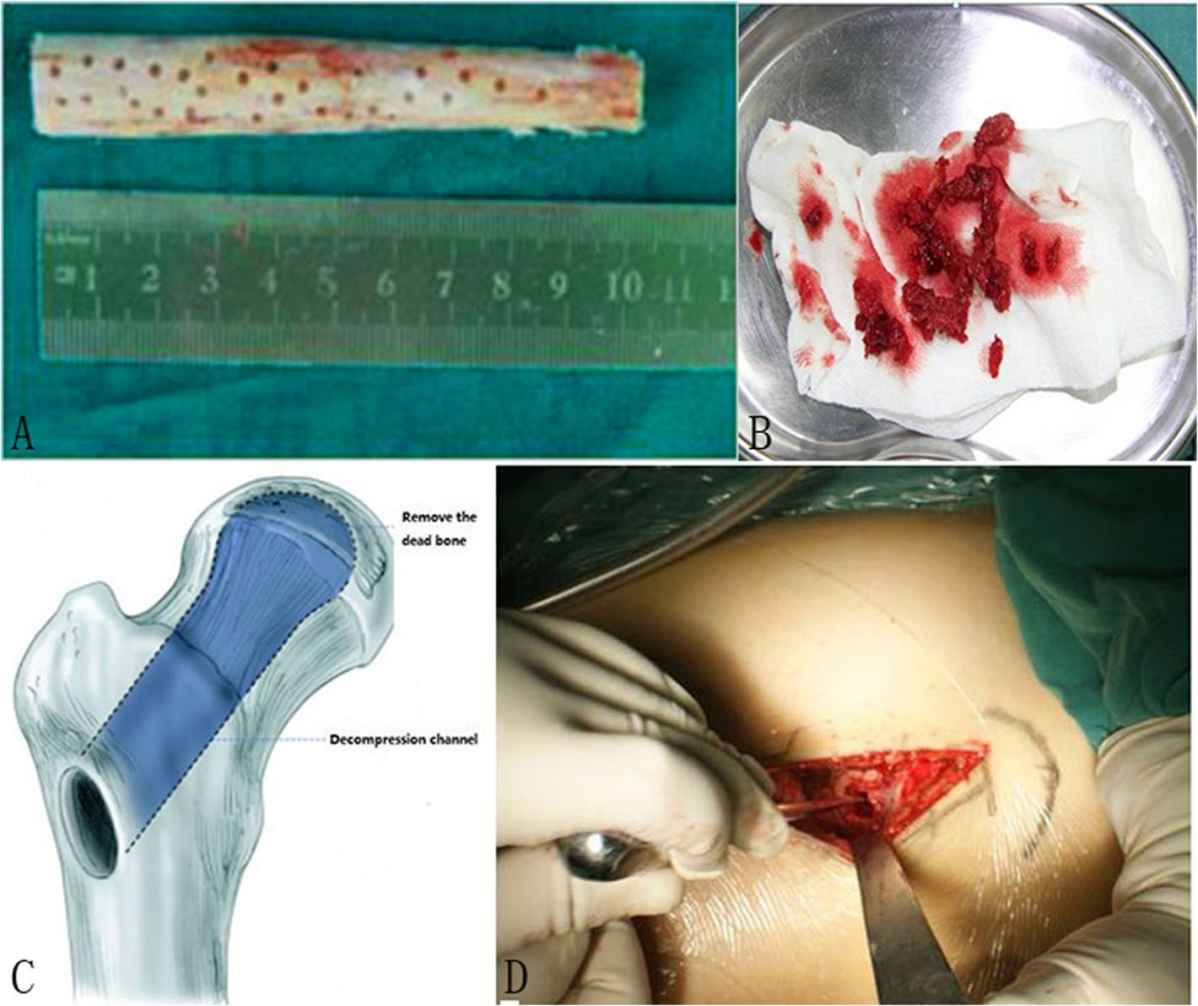
**a** The nonvascularized allogeneic fibula with 10 cm long and small holes on the surface. **b** Removed the dead bone through the decompression channel. **c**, **d** Diagrammatic drawing of the decompression channel. **c** Creating the decompression channel during the operation. **d** The channel has been built and an allogeneic fibula has been grafted

Before the surgery, non-vascularized allogeneic fibula was obtained from the bone bank of the hospital with the antigen removed by cryogenic freezing. Some holes were made around the fibula with a drill of 1 mm in diameter by the purpose of making it easier for the new bone tissue growing into the allogeneic fibula (Fig. 1).

All cases were performed by a single senior surgeon (PD Kang) using the same surgical technique: the decompression channel up to the subchondral bone and necrosis area was made by hollow trephine via the navigation of Kirschner wire under fluoroscopic control in the femoral head, which was used to drill a hole at area 6 cm below the femoral greater trochanter vertex and through the neck of the femur (Fig. 1). And then, removed the dead bone through the decompression channel with curet and sent for pathologic examination (Fig. 1). After that, the cancellous bone obtained from the area of the femoral trochanter through the decompression channel was compacted to the femoral head and then modified the form of the non-vascularized allogeneic fibula and grafted it to the channel. The fibula was knocked by a bone hammer to make it located tightly and the rest of autogenous cancellous bone was filled to the space around the grafted fibula. The distal end of the fibula should reach the area 5 mm below the surface of the femoral head. Fluoroscopy confirmed the adequacy of the positioning of the graft. After the procedure, conventional analgesic and thromboprophylaxis were conducted and functional exercise with active flexion and abduction of the surgery hip was performed. Walking with a crutch and light-touch weight-bearing were advised before the third post-operative month and full weight-bearing was permitted until radiographic evidence of graft healing. Strenuous activity should be avoided for a year.

### Outcome assessment

Follow-up examinations included clinical and radiological assessment were scheduled at 1, 4, and 12 months and then once a year. Visual analog scale (VAS) pain scores (VAS 0; no pain to 10; extreme pain), range of hip motion (ROM) and Harris hip score (HHS, the full score is 100 marks, 90–100 is excellent, 80–90 is good, 70–80 is ok and less than 70 is bad) was used to analyze the clinic outcome; Western Ontario McMaster Osteoarthritis index (WOMAC, including pain, stiffness, and physical function) scores and Short Form 36 health survey (SF-36) were conducted to measure the living quality; radiological assessment X-ray or computerized tomography (CT) scan help to evaluate the stage of the disease. MRI was not routinely used for the postoperative evaluation. The survivorship was equal to the total percentage of the patients who remained in good condition and did not need to undergo the THA or fusion.

### Statistical analysis

The SPSS 20.0 software was used for statistical analysis. The results were analyzed by the Student’s t test. A probability of less than 0.05 was considered statistically significant.

## Results

Patients were followed with the examination in our outpatient service. The postoperative follow-up of these 44 patients ranged from 6 to 8.2 years with an average of 7.4 years. The mean age of the patients at the time of surgery was 36.46 years (range, 26–47 years). There were 29 men and 15 women. Associated etiology factors included alcohol (26 patients), steroid use (15 patients), and idiopathic (3 patients). The radiographic appearance, according to the Ficat classification for ONFH,44 patients were at FicatII stage at the time of surgery. During the follow-up, 6 patients were lost for different reasons and 44 patients were included in this study. No serious complications, such as deep infection, fracture, and grafted bone dislocation, have happened.

### Clinical and health assessment

The majority of patients reported considerable relief of hip pain at night and better hip motion in daily activity and work. VAS scores at rest or activity were improved from 2.3 to 1.2 on average or from 4.0 to 2.8 on average, respectively. And the overall HHS scores were improved from 70.5 to 82.5 on average at the last follow-up, and 10 cases showed an excellent outcome, 18 patients were with good results, 11 cases had ok scores but 5 were bad. All of the range of motion (ROM) items at last follow-up, including flexion, abduction, adduction, external rotation, internal rotation, achieved significant improvement compared with pre-operation. The mean VAS score, ROM, and Harris hip score were significantly improved at final follow-up compared with preoperative values (*p* < 0.001), and health assessment items including WOMAC scores and SF-36 were also better than pre-operation (*p* < 0.001), details were shown in Table 2.

**Table 2:** baseline and outcomes of the last fellow-up

Variable	Pre-operation	Last follow-up	*p* values
Clinical outcomes			
VAS score			
At rest	2.3(1.5–2.8)	1.2 (0–3.4)	*p* < 0.001
With activity	4.0 (3.1–4.6)	2.8(1.9–4.8)	*p* < 0.001
Hip Harris score	70.5 (66–74)	82.5 (52–92)	*p* < 0.001
ROM (°)			
Flexion	90.8 (86.4–96.8)	104.5 (76.3–115.7)	*p* < 0.001
Abduction	31.1 (27.6–35.2)	38.5 (22.0–43.8)	*p* < 0.001
Adduction	16.6 (15.9–17.4)	22.3 (16.0–24.3)	*p* < 0.001
External rotation	30.2 (28.8–31.6)	39.1 (21.8–43.6)	*p* < 0.001
Internal rotation	11.2(10.1–12.7)	18.9(9.5–22.2)	*p* < 0.001
Health status			
WOMAC^a^			
Pain (0–20)	9.4 (5–11)	4.4 (2–12)	*p* < 0.001
Stiffness (0–8)	3.1 (0–4)	2.2 (0–3)	*p* = 0.002
Function (0–68)	44.6 (32–58)	28.3 (19–60)	*p* < 0.001
SF-36^b^			
General health	56.4 (47.0–66.0)	62.5 (45.0–74.0)	*p* < 0.001
Mental health	70.6 (65.0–74.0)	71.2 (58.0–80.0)	*p* = 0.122
Bodily pain	67.2 (60.0–73.0)	82.4 (60.0–90.0)	*p* < 0.001
Physical function	59.9 (46.0–65.0)	74.8 (48.0–86.0)	*p* < 0.001
Social function	65.4 (55.0–72.0)	76.9 (53.0–84.0)	*p* < 0.001

^a^WOMAC dimension scores range 0–20 (pain), 0–8 (stiffness) and 0–68 (physical function); a higher score indicates increased pain or stiffness or worse physical function

^b^SF-36 dimension scores range 0–100: a higher score for each dimension indicates the better health-related quality of life

### Radiographic outcomes

**Fig. 2:**
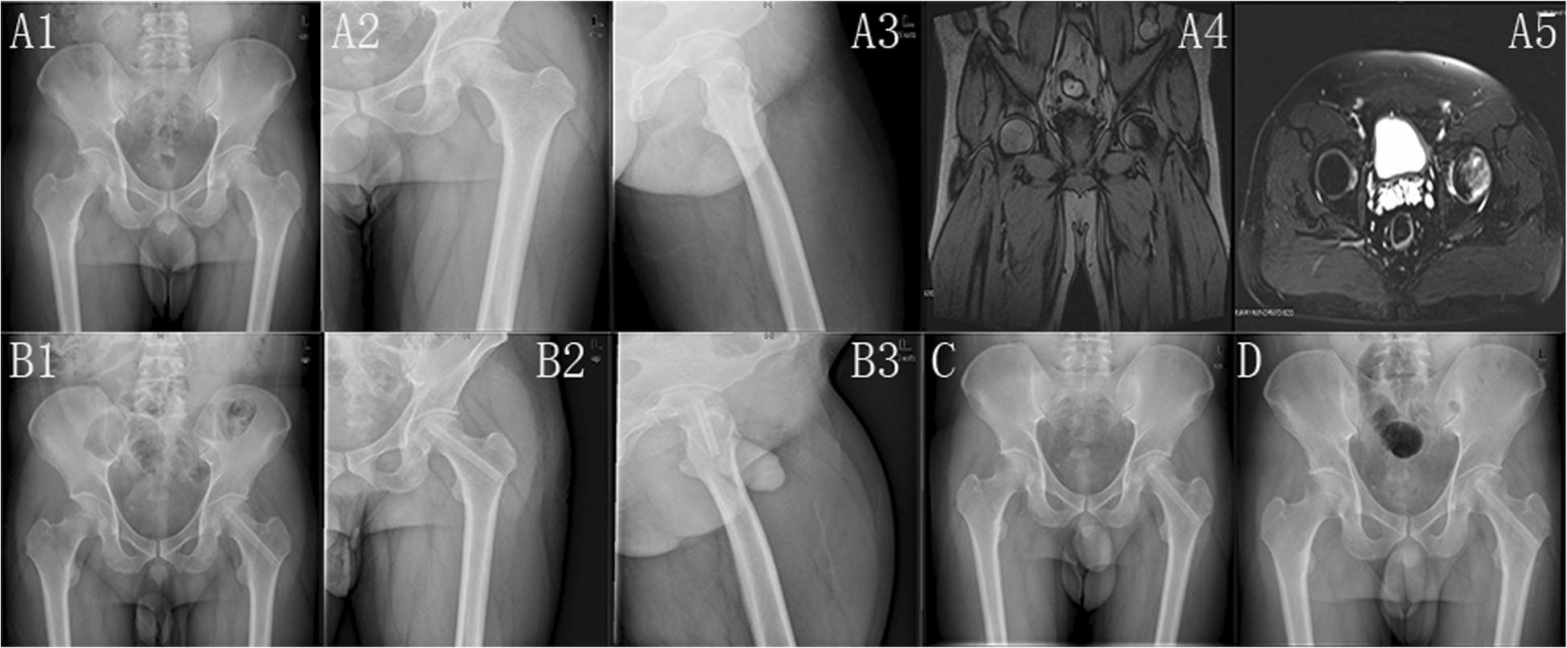
The radiographic outcomes of a 36-year-old man with a long history of drinking and was diagnosed as left femoral head necrosis at Ficat II stage. **A1**–**A5** The patient has early femoral head necrosis evaluated by X film and MRI respectively. **B1**–**B3** The X film of 3 days after the operation. It shows that the femoral head is in normal shape and the grafted fibula integrates tightly in the channel. **C**, **D** The images at 1 and 4 months after the operation, respectively. **E1**–**E3** The images at 2 years after the operation. **F1**–**F3** The images at 5 years after the operation, which shows the stability of the articular cartilage and femoral head. **G1**–**G3** The images at 8.2 years after the operation. The femoral head is not progressed and keeps in good shape. The left femoral head was in Ficat I stage and the Harris score was 90 at the last follow-up

**Fig. 3:**
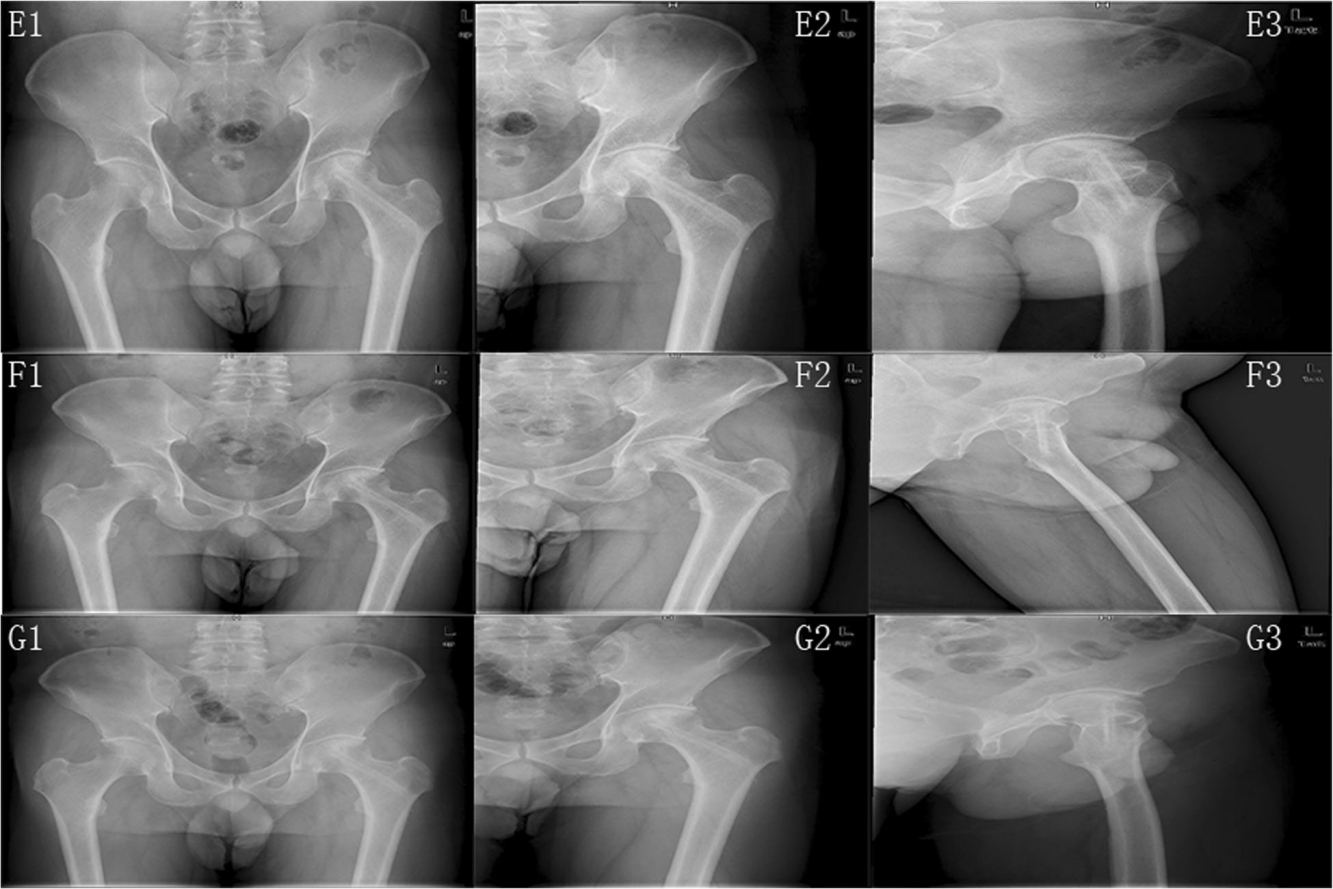
The radiographic outcomes of a 36 years old man with a long history of drinking and was diagnosed as left femoral head necrosis at Ficat II stage. **A1**–**A5** The patient has early femoral head necrosis evaluated by X film and MRI respectively. **B1**–**B3** The X film of 3 days after the operation. It shows that the femoral head is in normal shape and the grafted fibula integrates tightly in the channel. **C**, **D** The images at 1 and 4 months after operation respectively. **E1**–**E3** The images at 2 years after the operation. **F1**–**F3** The images at 5 years after the operation, which shows the stability of the articular cartilage and femoral head. **G1**–**G3** The images at 8.2 years after the operation. The femoral head is not progressed and keeps in good shape. The left femoral head was in Ficat I stage and the Harris score was 90 at the last follow-up

At the last follow-up, the radiographic outcome was improved in 5 patients with the classification of Ficat I and 28 patients remained unchanged. However, 11 patients had a radiographic progression, in which 7 patients progressed to Ficat III with the femoral head collapsed and the rest 4 progressed to Ficat IV with mild osteoarthritis. The 7.4-year radiographic progression rate was 25%. The representative case is shown in Figs. 2 and 3.

### Survivorship

There are 11 patients progressed and had the femoral head collapsed during follow-up time, 4 of whom had a good condition and the pain can be controlled after taking a small amount of painkiller without the gastrointestinal function being harmed. However, 8 patients who cannot tolerate the pain and had poor living quality underwent THA. The success rate of 7 years was 81.8%.

## Discussion

In this study, we evaluated the clinical results, health status, radiographic outcomes, and survivorship at a mid- and long-term follow-up after treating the early ONFH with modified non-vascularized allogeneic fibula grafting combined with core decompression and bone grafting. According to our outcomes, the clinical results evaluated with VAS score, HHS, the ROM, and health status assessed with WOMAC scores and SF-36 were improved significantly; 11 cases had radiographic progression with a rate of 25%; 8 patients had THA and the 7 years’ success rate was 81.8%.

In order to protect the femoral head from collapse and delay the THA, it is important to take effective and positive measures to protect the femoral head at the early stages of ONFH. Two key points must be considered. The first one, the biological factor, which included removing the dead bone adequately and promote bone regeneration and blood flow recovery. The other is biomechanics factor that is to implant the scaffold, including implantation bioactive materials or not, to function as mechanical supporting and osteoconduction to the defect area so that it can delay or prevent the femoral head from collapse [18, 19]. Though several studies have revealed that tantalum rod insertion can effectively delay or prevent the progression of osteonecrosis of the femoral head, whose overall survival rates were 68.1 to 72.49% at 48 to 60 months postoperatively [20, 21]. However, in our institution, we found a limited curative effect of tantalum rod implantation. In contrast with the disadvantages of transtrochanteric rotational osteotomy and tantalum rod placement, vascularized fibular graft with greater nutrient blood supply was preserved, osteocytes and osteoblasts remained, significantly enhance the biological and mechanical properties to the necrosis area, which exerting excellent effects in improving the clinical outcomes and survivorship [22–25]. As shown in Table 3, the middle follow-up outcomes were various with a success rate of 60 to 100%, which was encouraging. But complications often occur after the surgery, especially when the fibula is harvested, which may decrease the satisfactory degree of the patients. Free vascularized fibular grafting used for ONFH has been associated with proximal femoral fracture and donor-site morbidity including sensory abnormalities, motor weakness, and ankle pain, which might influence the recovery of the patients and prolong the rehabilitation [14]. In order to obtain the free vascularized bone, a 10 cm to 15 cm fibular is removed from the patients, leaving remnants of bone proximally and distally, which may increase the instability of the lower extremity, though mostly acceptable but should also take into account [30]. According to the previous studies, about one-fourth of the patients reported discomfort, pain or swelling, and sensory deficits in 76.3%, motor deficits in 39.5% and reduced strength in 44.7% were found in the included patients at the time of follow-up examination [31]. Moreover, free vascularized fibula graft surgery is complicated, thus, most of the community hospitals or less prosperous region think it is impractical to do such a surgery so that they continue to perform core decompression today [32]. Consequently, although the free vascularized fibular grafting has good effects on treating ONFH, potential complications and the cost-effective value are needed to be taken into consideration and more appropriate techniques should still be researched.

**Table 3:** Results of fibular grafting for the treatment of ONFH according to the literature

Reference	Year of publication	Number of hips	Treatment measures	Follow-up in months	Success rate (%)
Sotereanos [26]	1997	88	Free vascularized fibula graft	66 months	77.3
Berend [15]	2003	224	Free vascularized fibular grafting	60 months	64.5
Bertrand [16]	2013	52	Vascularized fibular graft	19 months	90.1
Gao [17]	2013	42	Free vascularized fibula graft	42 months	100
Buckley [27]	1991	20	Tibial or fibular autogenous graft,	24 months	95
Yin [22]	2011	14	Free vascularized fibular grafting	18 months	100
Lin [28]	2009	32	Allograft fibula grafting	36 months	93.8
Eward [23]	2012	65	Vascularized fibular graft, free vascularized fibular graft	96 months	60
Zhang [29]	2011	28	Vascularized fibular grafting	48 months	96
Kim [24]	2005	23	Non-vascularized fibular grafting	48 months	87
		23	Vascularized fibular grafting	48 months	78
Plakseychuk [25]	2003	220	Non-vascularized fibular grafting	84 months	86
		123		84 months	30

Currently, many studies have compared the safety and efficiency of vascularized and non-vascularized fibular grafting, most of which found that vascularized fibular grafting treatment have a better survival rate as well as better clinical and radiological results than non-vascularized fibular grafting treatment [25, 33]. However, different from the precious non-vascularized fibular grafting method, our modified one shows a satisfactory survival rate as well as clinical and radiological outcomes and decreases complexity and the occurrence of adverse events. In our study, we chose the non-vascularized polyporous allogeneic fibula from the bone bank of our hospital as the graft material. In order to increase the bioactivity, we grafted autogenic cancellous bone to the femoral head before the transplantation. According to our outcomes, our surgery significantly reduced the pain, increased the range of the motion and improved the HHS, which demonstrated that modified non-vascularized allogeneic fibula grafting combined with core decompression and bone grafting had effects on improving the clinical results. And then, at our mean 7.4 years follow-up, most of the patients were not progressed when evaluated by the radiographic examination and some of them even improved to the better classification. This helps to prove that our modified operation contributed to protecting the femoral head from becoming worse. Furthermore, the success rate of this study came to 81.8% and was not inferior to free vascularized fibular grafting based on the above studies. Moreover, there were no obvious complications that happened during our follow-up, and the patients achieved a faster rehabilitation and better living quality after operation for not removing the autogenous fibula that could prolong the operation time and hospital stays, increase the blood loss and local pain or dysaesthesia. When evaluating the health status, WOMAC and SF-36 showed improved outcomes in the living quality items, which include pain, stiffness, physical function, and social function. Besides, Zeng Y et al. [34] found that core decompression followed by bone grafting combining with non-vascularized fibular allografting in one hip and concurrent one-stage total hip arthroplasty (THA) in the contralateral side is efficient for the treatment of bilateral osteonecrosis of the femoral head in the short term, which can provide biological stability and sufficient blood supply and obtain longtime repairmen of the necrotic bone. Combined with previous studies and our outcomes, we may prove that patients with ONFH have good tolerance to this modified surgery in the mid- to long-term, and can obtain a quick physical function recovery and high satisfaction of living.

Normally, a combination of allogeneic and autologous bone transplants is a reliable method to biologically reconstruct bone defects, of which autologous cancellous bone is well-known for its osteogenic potential; and combined therapy can provide desirable safe load transmission and osteoinduction and remodeling [35, 36]. In the present study, allogeneic-fibular from the bone bank remains the same structures as an autogenous bone with antigen removal could be an effective supporting material to the subchondral architecture. At the same time, the autogenic cancellous bone can function as a good structure, which might facilitate mesenchymal stem cells associated with bone regeneration, adding the osteogenic activity to the necrosis area and benefiting the repair of the defects [37]. However, several limitations were obvious in our study. First, we only included 44 hips in our research and our outcomes may not persuasive enough yet. Second, the allogeneic-fibular is very limited in clinical, so it is necessary and important to build a bone bank. Third, our follow-up period is still not long enough and longer-terms of follow-up are still needed to prove our results. Fourth, we failed to include advanced stages of ONFH, it is imperative to estimate the curative effect of our method in these patients.

In conclusion, modified non-vascularized allogeneic fibula grafting combined with core decompression and bone grafting exerted is an effective and cost-effective treatment on early femoral head necrosis with satisfactory survivorship and could improve the clinical outcomes, delay the disease progression, and enhance the quality of life for patients.

## Data Availability

The datasets used and/or analyzed during the current study are available from the corresponding author on reasonable request.

## Abbreviations

HHS: Harris hip score
ONFH: Osteonecrosis of femoral head
ROM: Range of hip motion
SF-36: Short Form 36 health survey
THA: Total hip arthroplasty
VAS: Visual analog scale
WOMAC: Western Ontario McMaster Osteoarthritis index
